# Biocatalytic Performance of *β*-Glucosidase Immobilized on 3D-Printed Single- and Multi-Channel Polylactic Acid Microreactors

**DOI:** 10.3390/mi15020288

**Published:** 2024-02-18

**Authors:** Andreas-Georgios Vasios, Anastasia Skonta, Michaela Patila, Haralambos Stamatis

**Affiliations:** Laboratory of Biotechnology, Department of Biological Applications and Technologies, University of Ioannina, 45110 Ioannina, Greece; avasios1998@gmail.com (A.-G.V.); a.skonta@uoi.gr (A.S.); mpatila@uoi.gr (M.P.)

**Keywords:** 3D printing, microreactors, polylactic acid, *β*-glucosidase, enzyme immobilization, multi-channel parallel microreactors

## Abstract

Microfluidic devices have attracted much attention in the current day owing to the unique advantages they provide. However, their application for industrial use is limited due to manufacturing limitations and high cost. Moreover, the scaling-up process of the microreactor has proven to be difficult. Three-dimensional (3D) printing technology is a promising solution for the above obstacles due to its ability to fabricate complex structures quickly and at a relatively low cost. Hence, combining the advantages of the microscale with 3D printing technology could enhance the applicability of microfluidic devices in the industrial sector. In the present work, a 3D-printed single-channel immobilized enzyme microreactor with a volume capacity of 30 μL was designed and created in one step via the fused deposition modeling (FDM) printing technique, using polylactic acid (PLA) as the printing material. The microreactor underwent surface modification with chitosan, and *β*-glucosidase from *Thermotoga maritima* was covalently immobilized. The immobilized biocatalyst retained almost 100% of its initial activity after incubation at different temperatures, while it could be effectively reused for up to 10 successful reaction cycles. Moreover, a multi-channel parallel microreactor incorporating 36 channels was developed, resulting in a significant increase in enzymatic productivity.

## 1. Introduction

Enzymes comprise a valuable tool in various industrial applications since their recruitment provides significant advantages compared to chemical processing methods, such as low toxicity, high product specificity, mild reaction conditions, and biodegradability [1]. In this framework, *β*-glucosidases are enzymes of particular importance in the food, health, and bioenergy industries. More specifically, *β*-glucosidases belong to the wide class of hydrolases and can cleave the *β*-1,4 bonds of oligosaccharides with the spontaneous release of glucose molecules [1,2,3]. Different microorganisms can produce these enzymes, such as the thermophilic marine eubacterium *Thermotoga maritima*. The recombinant *β*-glucosidase that derives from this kind of organism can withstand high reaction temperatures and can metabolize a variety of substrates [3]. *β*-Glucosidases can find application in different biotechnological processes such as the food industry for the enhancement of the flavor of certain foods and beverages and detoxification of foods [2], the pharmaceutical industry for the release of bioactive compounds [2], and biofuel production for the conversion of cellulosic compounds to glucose [4].

Enzyme immobilization can greatly enhance the performance of the biocatalysts in the industrial sector, allowing for greater stability and the possibility of reusing them multiple times without needing purification [1]. In some cases, to further enhance the productivity of immobilized enzymes, microfluidic systems can be used [5]. Microfluidic devices have been widely used in research owing to their beneficial characteristics, especially in enzymatic biotechnology where precise condition control is required to achieve desirable results [6]. In detail, the microscale grants these systems high surface-to-volume ratios, enhanced mass and heat transfer rates, and precise control of the flow type [7]. Hence, higher enzymatic activity and measurement precision may be achieved as well as low reagent consumption and short reaction times [8,9]. Nevertheless, microfluidic devices also possess several disadvantages, such as the need for specialized equipment, high production cost and time, and difficulty in scaling-up [10,11]. Concerning the scaling-up process, the two main methodologies that can be followed are known as numbering up and sizing up; numbering up is based on the parallelization of several conventional microreactors, whereas sizing up is focused on increasing the capacity of a single microreactor to improve productivity [5,12].

Many of the above restrictions could be overcome by the three-dimensional (3D) printing technology, which during the past 20 years has been proven to be a robust tool for scientists, especially in the field of biotechnology [13]. Briefly, the manufacture of 3D-printed models is divided into three separate and consecutive steps: design, slicing, and printing. The designing step determines the shape of the model, the slicing step sets the printing parameters and methodology, and the printing step manufactures the 3D model as dictated by the previous steps [8]. In some cases, a post-processing step is required to improve the surface functionality of the 3D-printed material [14]. The whole procedure allows for continuous adjustment and precise control over the quality of the printed model. Furthermore, the low cost of acquisition and operation of a 3D printer combined with the lack of need for specialized personnel render 3D printing a promising solution for otherwise tedious procedures for device modeling [15,16,17].

Several examples of immobilized enzymes on 3D-printed scaffolds can be found throughout the literature. Nonetheless, these scaffolds are mainly used in biomedical applications and especially in the fields of drug delivery, implant manufacturing, and tissue engineering, where 3D printing has shown promising results [18,19]. Regarding microfluidic devices, 3D printing has been used to develop biosensing elements for various biomarkers such as glucose, lactate, and glutamate [14,20]; however, microfluidic devices have been used for biocatalytic purposes as well. In more detail, enzymes like ω-transaminases, proteases, laccases, and lipases have been successfully immobilized and utilized in such devices to catalyze the biotransformation of a variety of substrates such as phenols and ketones [21,22,23,24]. It is worth noting that 3D printing has been used to produce microfluidic devices through the techniques of stereolithography (SL), binder jetting (BJ), and material extrusion (ME), contributing to the reduction in production cost and time as well as the easy customization of the printing process [10]. These 3D printing techniques utilize a variety of compounds as printing materials, ranging from photopolymer resins and thermoplastic materials to composite materials, each with its own characteristics and advantages. This makes 3D printing capable of being applied in many different fields. Among them, industrial production has attracted much attention [25]. Many of the materials used in 3D printing, such as polylactic acid (PLA), are biocompatible and biodegradable, making the devices manufactured ecologically friendly and thus reducing the ecological footprint of the industries that utilize them [10,26,27]. However, PLA lacks the necessary reactive groups that are required for enzyme immobilization. This limitation can be overcome through surface modifications [28]. For instance, chitosan, a natural polymer that contains plenty of amine groups in its structure, can be grafted on PLA via electrostatic interactions, providing the surface of the 3D-printed model with reactive groups that can be used for enzyme immobilization [29].

In the present work, two 3D-printed devices were designed and fabricated in one step via the fused deposition modeling (FDM) printing technique using PLA as the printing material. A single-channel microreactor with a volume capacity of 30 μL and a multi-channel parallel microreactor incorporating 36 channels were created. Both devices had to undergo surface modification with chitosan for the immobilization of enzymes to be possible. The single-channel device was used to investigate the biocatalytic properties of immobilized *β*-glucosidase from the microorganism *Thermotoga maritima*. In addition, a scaling-up process of this microfluidic system was attempted using the multi-channel parallel device.

## 2. Materials and Methods

### 2.1. Materials

Polylactic acid (PLA) filament with a diameter of 1.75 mm was obtained from Prima Creator (Malmo, Sweden). Sodium hydroxide (>98%) was purchased from Panreac (Barcelona, Spain). Chitosan from crab shells (85% deacetylated), acetic acid (>99.8%), p-nitrophenyl-*β*-D-glycopyranoside (p-NPG), and p-nitrophenol (p-NP) were obtained from Sigma-Aldrich (Germany). *β*-Glucosidase from *Thermotoga maritima* was obtained from Megazyme (Chicago, IL, USA). Glutaraldehyde (25% solution) was purchased from Fisher Scientific (Hampton, NH, USA).

### 2.2. Design and Fabrication of the 3D Scaffolds

**Figure 1:**
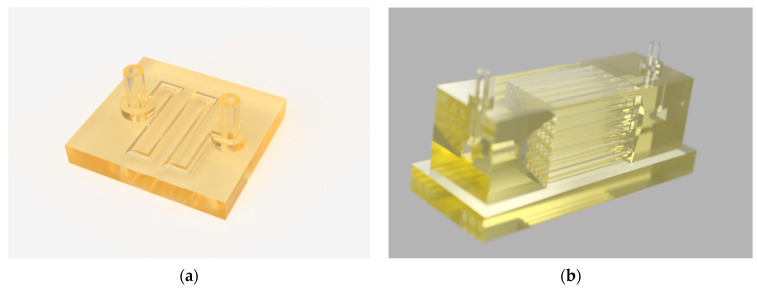

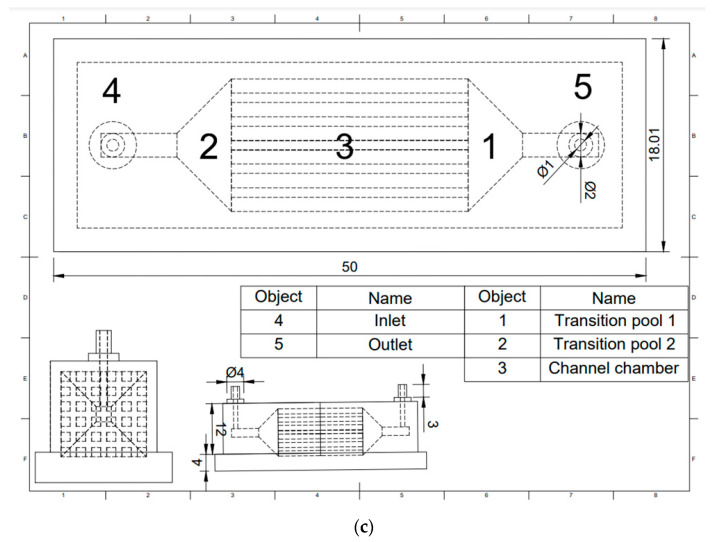
Digital representations of (**a**) the single-channel microreactor and (**b**) the multi-channel parallel microreactor system. Pictures of both reactors were taken after rendering in Fusion 360 (Autodesk) based on the original CAD files. (**c**) Schematic representation of the multi-channel microreactor. The schematic was generated using the Autodesk “Design” feature based on the original design (dimensions in mm).

The 3D computer-aided (CAD) models were designed in the 3D modeling environment of the Fusion 360 software (Autodesk). Two scaffolds were created for this work: a single-channel microreactor with a capacity of 30 μL and a multi-channel parallel microreactor (Figure 1). The single-channel microreactor consisted of a circular channel with an internal diameter of 0.8 mm. The channel was designed in a curvilinear pattern to render the printed structure more compact. The model also incorporated an inlet and an outlet. The multi-channel parallel microreactor was designed with a total capacity of 1.52 mL. The design consisted of three modules, two transition modules (0.45 mL), one parallel microreactor module (0.72 mL), an inlet, and an outlet with diameters of 1 mm. The transition modules guarantee a smooth transition from the inlet and outlet flow to and from the parallel reactor module. The parallel reactor module consisted of 36 rectangular channels with a width and length of 1 mm, while its height was 20 mm.

The slicing procedure was performed in the Cura slicing software (Ultimaker). The printing settings for each model were 0.12 mm layer height (*z*-axis resolution), 100% infill density, and 60 mm/s print speed. The printing temperature was set to 200 °C and the build plate temperature was set to 50 °C. The designed models were printed via the FDM technology on the 3D printer Ender 5 (Creality 3D, China), which was fitted with a 0.4 mm brass nozzle. No modifications or extensions were added to the printer.

### 2.3. Surface Modification of the PLA Scaffolds

The surface modification of the PLA scaffolds consists of two stages: the etching of the internal surface of PLA and its functionalization with chitosan [29]. Regarding the etching process, a 1 M sodium hydroxide (NaOH) solution was used. The solution was incubated in the interior of the PLA scaffolds for 2 h at 25 °C. The NaOH solution can hydrolyze the PLA chain at its ester bonds, revealing carboxyl and hydroxyl moieties so that chitosan can be grafted in the following step [30]. A 0.2% *w/v* chitosan solution was prepared using an aqueous solution of 1% *v/v* acetic acid as a solvent, which was then incubated with the PLA scaffolds for 1 min at 25 °C. To ensure the neutralization of the pH, the PLA scaffolds were re-incubated with a 1 M NaOH solution for 1 min. Between the different modification stages of the PLA surfaces, the scaffolds were washed with ddH_2_O, and any excess moisture was removed under the N_2_ stream.

### 2.4. Immobilization of β-Glucosidase in the Microreactors

The enzyme immobilization in the single-channel microreactor was achieved through covalent attachment using glutaraldehyde as the cross-linker. At first, the modified PLA surfaces were incubated with varying concentrations of glutaraldehyde solutions (0.25–5.0% *v/v*) in phosphate buffer (pH 7.0, 50 mM) for 1 h at 30 °C. Washing with the buffer solution and drying followed as in the previous steps. The PLA surfaces were then incubated for 1 h at 30 °C with aqueous solutions of *β*-glucosidase from *Thermotoga maritima* in 100 mM phosphate buffer pH 6.5 (0.25–4 μg/mL final concentration). Lastly, the PLA scaffolds were washed with the buffer solution (phosphate buffer pH 6.5, 100 mM) and dried under a N_2_ stream. For the immobilization of the enzyme in the multi-channel parallel microreactor, the same procedure was followed except the inserted volume was adjusted to match the capacity of the reactor and the concentration of the enzyme solution was 2 μg/mL. All experiments were performed in duplicate.

### 2.5. β-Glucosidase Activity Studies

The enzymatic activity of the free *β*-glucosidase was measured using p-NPG as the substrate. *β*-Glucosidase hydrolyzes the β-1,4 bond of the p-NPG to produce p-NP and glucose. The p-NPG hydrolysis reactions were conducted in 96-well ELISA plates in a final volume of 150 μL. The reaction solution contained 1 mM p-NPG and 0.1 μg/mL *β*-glucosidase, both dissolved in 100 mM of phosphate buffer at pH 6.5. The reaction was performed at 40 °C for 10 min and the absorbance of the produced p-NP was measured at 405 nm.

The enzymatic activity of the immobilized *β*-glucosidase in the single-channel microreactor was also determined by the p-NPG assay. In this case, a 1 mM p-NPG solution in phosphate buffer (100 mM, pH 6.5) was pumped through the microreactor at a flow rate of 1.8 mL/h using an SP200 or an SP100 Series Syringe Pump (World Precision Instruments, Ltd., Hertfordshire, UK) with Tygon tubing, which corresponds to a residence time of the solution in the microreactor of 1 min. The microreactor, as well as part of the tubing and the collection vials, was placed inside an incubator with a preset temperature of 40 °C. At 5-minute intervals, 150 μL of effluent was collected and transferred to an ELISA plate, and its absorbance was measured at 405 nm. One reaction cycle was completed when 150 μL of the solution passed through the microreactor. Moreover, the effect of pH was studied by measuring the activity of both free and immobilized *β*-glucosidase at pH values ranging from 5.0 to 9.0.

The enzymatic activity of the immobilized *β*-glucosidase in the multi-channel parallel microreactor was measured with the p-NPG assay as described above with some adaptations. In this case, a 1 mM p-NPG solution in phosphate buffer (100 mM, pH 6.5) was pumped through the microreactor at 40 °C and a flow rate of 9 mL/h, which is equivalent to a residence time of 2.25 min in the reactor module of the device using a BT100-1L Multi-channels Peristaltic Pump (Longer Precision Pump Co., Ltd., London, UK). For the absorbance measurement of the product, 150 μL of effluent was transferred to an ELISA well plate and its absorbance was measured at 405 nm.

### 2.6. Kinetic Studies of β-Glucosidase

For the determination of the Michaelis kinetic constant (Km) of the free enzyme, 10 μL of a 0.2 μg/mL enzyme stock solution in phosphate buffer (100 mM, pH 6.5) was added to 190 μL of p-NPG of different final substrate concentrations (0.25–4 mM). Finally, the absorbance of the solution was measured at 405 nm at 30 s intervals for 10 min.

In the case of the immobilized *β*-glucosidase in the single-channel microreactor, the enzymatic activity of the immobilized enzyme was measured by using different initial substrate concentrations (0.25–4 mM) and various flow rates ranging from 20 to 50 μL/min. For the estimation of the apparent Km of the immobilized enzyme, the Lilly–Hornby equation was used (Equation (1)):

(1) $$F\times {\left[A\right]}_{0}=K_{m\left(app\right)}\times \ln\left(1-F\right)+\frac{C}{Q}$$

where F is the fraction of the substrate converted to the final product during the reaction, Q is the flow rate of the substrate, [A]_0_ is the initial substrate concentration, C is the reaction capacity of the microreactor, and Km(app) is the apparent Michaelis constant. The Lilly–Hornby model is an adaptation of the Michaelis–Menten model that was initially created to describe enzyme kinetics in packed bed columns [31]. Later, the same equation was also found to apply to microreactors as [32].

### 2.7. Thermal and Operational Stability of β-Glucosidase

The thermal stability of the immobilized enzyme in the single-channel microreactor was evaluated for 24 h, after incubation of the microreactor at 30, 40, and 50 °C in a thermostatically controlled incubator (INCU-SHAKER™ MINI, Benchmark Scientific, Sayreville NJ, USA). At the end of the incubation time, the enzymatic activity was remeasured to evaluate the residual activity of the immobilized enzyme, as described above.

The operational stability of the immobilized *β*-glucosidase was evaluated by measuring the activity of the enzyme after consecutive reaction cycles, at 40 °C. For both the single-channel and multi-channel microreactors, the reaction cycle was set as the time it takes for them to produce 150 μL of product solution.

### 2.8. Computational Fluid Dynamics Simulation

The computational fluid dynamics (CFD simulation) simulation was executed using the CFD software provided by Autodesk (Version 23.1). In the CFD program, the simulation model was imported from Fusion and meshed with the auto-meshing tools of CFD. The boundary conditions were set as 0–250 μL/min at the inlet and as zero pressure to the outlet. The solution of the simulation was set as a transient simulation and featured a linear increase in flow rate of 10 μL/min with each passing step of the process. Water was the liquid used for the simulation, while the simulation process took place using the default initial condition parameters of the software. Results were calculated for each step in three iterations and the results were saved after every step was complete.

### 2.9. Statistical Analysis

All analyses corresponding to the single-channel microreactor were carried out in duplicate. Results are means ± SD of two independent experiments (*n* = 2). One-way ANOVA analysis and Tukey’s and/or Dunnett’s multiple comparison tests were implemented using IBM SPSS Statistics version 21 (SPSS Inc., Chicago, IL, USA).

## 3. Results

**Figure 2:**
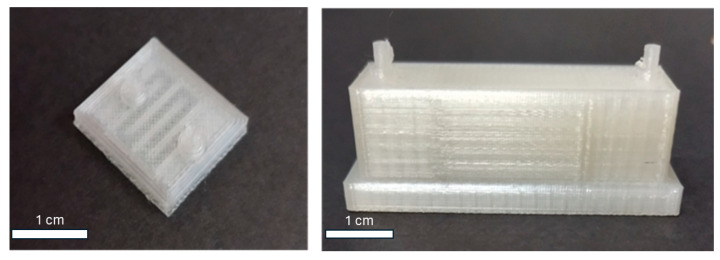
A 3D-printed single-channel microreactor (**left**) and a 3D-printed multi-channel parallel microreactor (**right**) for the immobilization of *β*-glucosidase.

In the present work, a 3D-printed single-channel microreactor was designed for the immobilization of *β*-glucosidase and the further estimation of its biocatalytic characteristics (Figure 2, left). This device features a compact size to minimize printing times and costs. Additionally, its geometric configuration that takes advantage of the limited size of the printing platform enables the user to print many reactors in one single printing step. In the next step, we designed a multi-channel parallel microreactor based on a design philosophy in which the entirety of the reactor is printed in one step, thus, simplifying the printing process (Figure 2, right). At this time, 12 reactors could be printed in parallel, and it is possible to increase the reactor number per printing process further. Its small capacity allows for the small consumption of reagents. Furthermore, smaller volume requirements make it possible for more test reactions to take place.

### 3.1. Optimization of the Immobilization Procedure in the Single-Channel Microreactor

**Figure 3:**
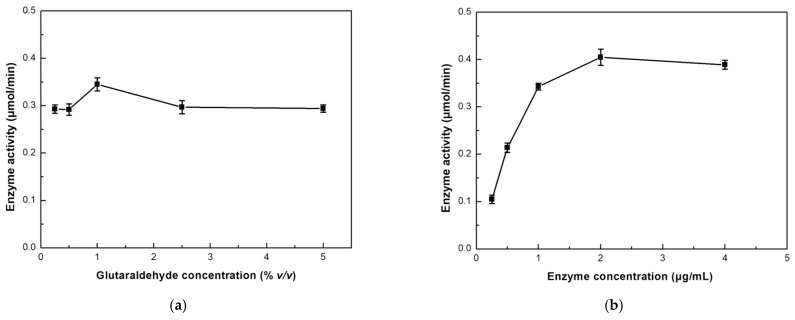
Effect of (**a**) glutaraldehyde and (**b**) enzyme initial concentration during the immobilization procedure on the catalytic activity of the immobilized *β*-glucosidase in the single-channel microreactor.

For the optimization of the immobilization process in the single-channel microreactor, two parameters were investigated: glutaraldehyde and enzyme concentration. Glutaraldehyde is a commonly used cross-linker that can connect the amine groups on a supporting material surface and the amine groups on an enzyme surface, by the formation of a Schiff’s base [33]. Thus, glutaraldehyde was used as a cross-linker for the covalent immobilization of *β*-glucosidase on chitosan. The proportion of glutaraldehyde during the immobilization procedure is critical; thus, different glutaraldehyde concentrations were studied, and the results are depicted in Figure 3a. As seen, the best glutaraldehyde concentration was found to be 1% *v/v* which provided a 15% relative increase in the enzymatic activity compared to lower or higher concentrations. Large concentrations of glutaraldehyde may result in lower enzymatic activity due to interactions of glutaraldehyde with important amino acid residues for the function of the enzyme. Furthermore, multiple bonds between chitosan and the enzyme may result in restriction effects that further lower the catalytic capabilities of the enzyme [34]. On the contrary, low concentrations of the cross-linker may result in insufficient immobilization of the enzyme [35]. The 1% *v/v* glutaraldehyde concentration was chosen as the optimal.

Enzyme consumption is an important economic factor in the manufacturing of microfluidic devices, and the excessive use of enzymes elevates the cost of this process, something that generally is to be avoided. To minimize the biocatalyst while maximizing the productive output of the device, we proceeded to investigate the effect of the enzyme concentration loaded on the microreactor on the activity of the immobilized enzyme. Figure 3b shows that the increase in enzymatic activity has a logarithmic correlation with the enzyme concentration in the concentration range of 0.25–2.0 μg/mL. However, when the enzyme concentration is further increased, the enzymatic activity starts to decrease. It has been previously reported that excess quantities of the enzyme may result in a reduction in the activity rate [36]. The microreactor interior may be saturated with enzyme molecules when higher enzyme concentrations are loaded, thus resulting in enzyme inactivation, steric hindrance, or mass transfer limitations. A similar result was reported when *β*-glucosidase was immobilized on zinc oxide (ZnO) microreactors [1] or when lipase from *Candida antarctica* (CaLB) was immobilized in a 3D-printed PLA microreactor [37]. Therefore, we propose the 2 μg/mL concentration as the optimal one to maximize biocatalyst efficiency.

### 3.2. Biocatalytic Characterization of the Immobilized Single-Channel Microreactor

**Figure 4:**
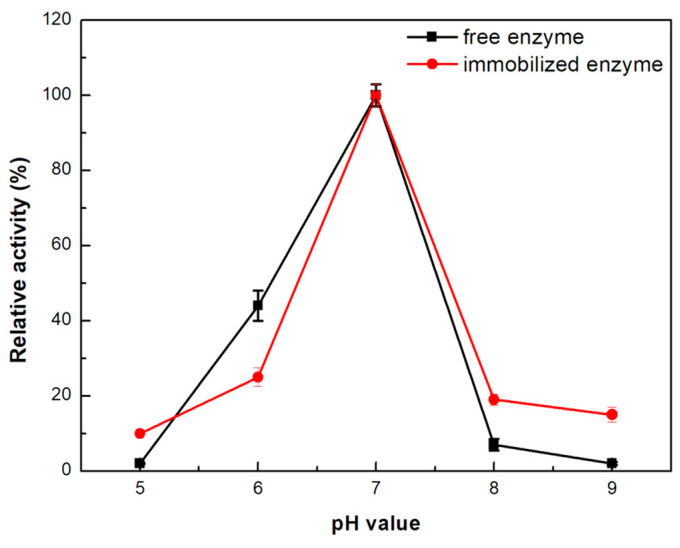
Effect of pH on the catalytic activity of the free and immobilized *β*-glucosidase in the single-channel microreactor. The value of 100% indicates the maximum observed activity at pH = 7.0.

The effect of pH on the activity of immobilized *β*-glucosidase in the single-channel microreactor was studied and compared to that of the free enzyme (Figure 4). Both enzyme forms exhibit maximum activity at a pH value of 7.0 like that previously reported in the literature [38,39,40,41,42,43]. When the pH value rises to 8.0 and 9.0, the immobilized enzyme excels in activity preservation over the free form, indicating a stabilizing effect of the microreactor towards the protein molecules [41,42]. A similar observation was previously reported for *β*-glucosidase immobilized in a continuous flow microreactor, where the immobilized microreactor exhibited greater tolerance to high pH values than the free enzyme [43].

**Figure 5:**
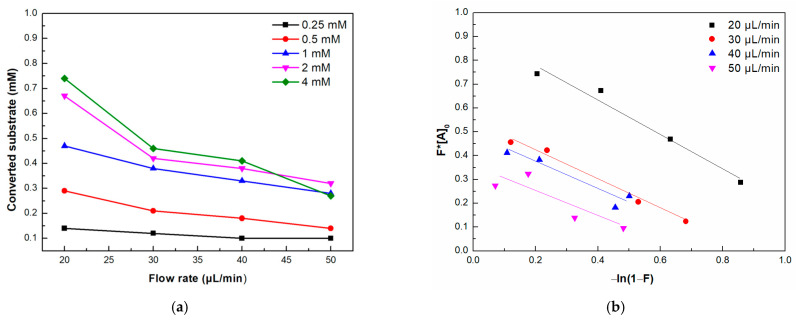
(**a**) Effect of the flow rate on the substrate conversion at different initial substrate concentrations and (**b**) use of the Lilly–Hornby model on the data collected from different flow rates and initial substrate concentrations.

To deepen our understanding of the catalytic performance of the immobilized enzyme in the single-channel microreactor, the effect of the flow rate on its activity was evaluated. The effect was studied regarding flow kinetics by applying the Lilly–Hornby model. Figure 5a shows the effect of the flow rate on the substrate conversion for initial substrate concentrations of 0.25–4.0 mM. It can be observed that lower flow rates result in higher enzymatic activity and thus higher substrate conversion. This could be explained by the residence time between the enzyme and substrate; as the flow rate decreases, the enzyme and the substrate have more time to interact with each other, leading to higher conversion yields, and vice versa [44]. Similar observations have been reported in other works involving the application of microfluidic devices [43,45,46,47].

In the next step, the apparent Km values were estimated using the kinetic data obtained at different initial substrate concentrations and flow rates. The linear plots of F*[A]_0_ versus *−ln(*1 *− F)* are presented in Figure 5b, where the slope corresponds to the Km(app). As seen from Table 1, the increase in the flow rate in the microreactor results in a decrease in the apparent kinetic constant of the immobilized enzyme. The Michaelis–Menten constant is generally associated with the affinity of the substrate to the enzyme; a decrease in the Km values arises from a higher affinity of the enzyme towards the substrate, while an increase in the Km values is correlated with a lower affinity between enzyme and substrate [48]. Moreover, in the case of immobilized continuous flow microreactors, the Km(app) is correlated with mass transfer phenomena; an increase in the Km(app) is associated with the presence of mass transfer limitations, while a decrease in the Km(app) is associated with the absence of a mass transfer effect [49]. Usually, the Km(app) value increases with the flow rate [50]. For instance, the Km(app) of immobilized naringinase in a microchannel reactor for the enzymatic synthesis of isoquercitrin was found to increase with an increasing flow rate, indicating significant mass transfer phenomena [51]. In another work, where *β*-glucosidase was immobilized in a silica quartz capillary tube, a 2.8-fold increase in the Km(app) was observed for flow rates increased in the range of 1–20 mL/min, suggesting that the enzymatic conversion rate of cellobiose was affected by mass transfer phenomena [44]. On the contrary, in our case, the Km(app) decreases with increasing flow rates, indicating a suitable affinity between the enzyme and the substrate and a restriction of mass transfer limitations. This result proves the effectiveness of the microreactor in promoting substrate diffusion toward the active site of the immobilized enzyme. A similar observation was reported by Bellou et al., where lipase from *Candida antarctica* was immobilized in a 3D-printed PLA tubular microfluidic reactor both in the presence or the absence of deep eutectic solvents (DESs) [37]. The authors reported that in the absence of DESs, the Km(app) of the enzyme-immobilized microreactor slightly decreased with increasing flow rate, suggesting that the system does not suffer from diffusion limitations. Other studies also report similar results [47,52,53]. It should also be noted that when moving from the 20 to the 30 μL/min flow rate, a significant decrease in the Km(app) value is observed, while from the 30 to the 50 μL/min flow rate, the differences in the Km(app) values are not considered statistically significant.

**Figure 6:**
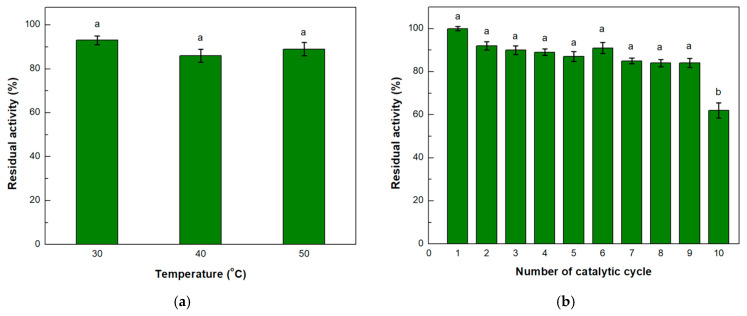
(**a**) Thermal stability and (**b**) operational stability of immobilized *β*-glucosidase on the single-channel microreactor. The value of 100% indicates the enzyme activity at t = 0 and in the first catalytic cycle for thermal and operational stability, respectively. Means with different letters are statistically significant (*p* < 0.05).

Thermal stability is a measure of evaluating the ability of an enzyme to preserve its three-dimensional structure and therefore its ability to function. To conduct this evaluation, the reactors containing the immobilized enzyme were incubated for 24 h at different temperatures. The results showed that the enzyme successfully retained over 80% of its activity in all temperatures ranging from 30 to 50 °C (Figure 6a).

One major advantage of immobilized enzymes is the ability to reuse them for multiple reaction cycles [1]. In the case of microreactors, where it is possible to provide the device with more substrate continuously, a reaction cycle is completed when a portion of the substrate solution passes through the entirety of the device; in the present work, the duration of the reaction cycle was 5 min. This device could operate continuously for ten consecutive reaction cycles while retaining above 80% of its initial activity until the ninth cycle (Figure 6b). These results are to be expected as this kind of system has proven to remain stable for many reaction cycles [39,41,54].

### 3.3. Development of a Multi-Channel Microreactor

**Figure 7:**
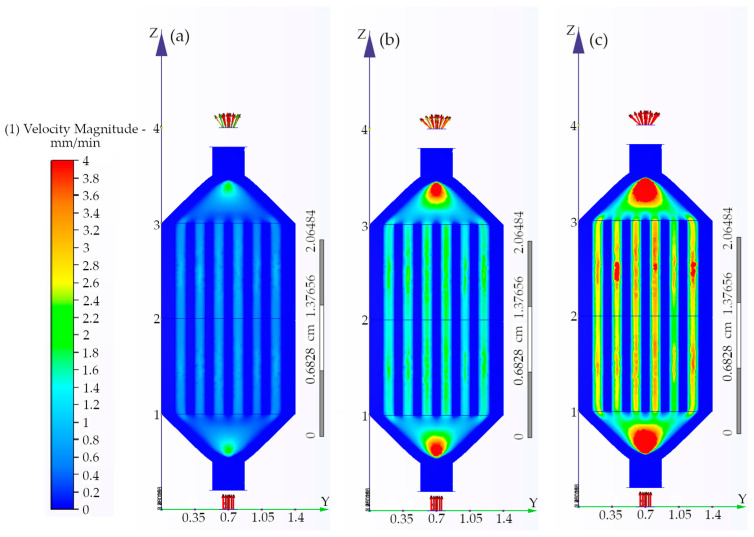
Schematic representation of flow distribution in the multi-channel parallel microreactor based on CFD simulations by the CFD software (Autodesk): (**a**) 50 μL/min, (**b**) 150 μL/min, and (**c**) 250 μL/min.

In the present work, we further proceeded with the development of a multi-channel 3D-printed enzyme microreactor with immobilized *β*-glucosidase. The preparation of this microfluidic system was achieved using an approach that simplifies the process by manufacturing the whole device in a single printing step, hosting 36 channels. To ensure the uniform distribution of the liquid, two flow transition pools were introduced in the device (Figure 2, right). The microdevice was modified with chitosan, and *β*-glucosidase was later covalently immobilized as previously described. The ability to predict the flow behavior inside the multi-channel device is of great importance in scaling up the process of microfluidic devices. A major challenge for the successful scale-up process is to ensure that the flow of the liquid is uniform. Therefore, the flow behavior of water was simulated at different flow speeds using the CFD software which has been widely used for this purpose [55,56,57]. The flow of the liquid was simulated for 125 different flow rates ranging from 2 to 250 μL/min. The simulation for three flow rates is presented in Figure 7. The flow of the liquid had turbulent characteristics, which is common for microfluidic devices with channels of greater width than 500 μm [58]. The flow of the liquid inside the channels was homogeneous up to the 150 μL/min flow rate, but further increases in flow speed resulted in major differences in the flow speed of individual channels of the device. For this reason, a threshold was set for this device at 150 μL/min.

In the following step, we used the above configuration for the covalent immobilization of *β*-glucosidase. The multi-channel microreactor was applied for the hydrolysis of p-NPG towards the production of p-NP. With this enzymatic multi-channel microreactor, we achieved a p-NP production of 0.074 μmol/min, which is 4.6-fold higher than that observed in the single-channel microreactor (0.016 μmol/min for the single-channel microreactor). It is worth noting that further optimization of the enzymatic microreactor performance could be achieved through CFD simulations as indicated in the work of Venezia et al. regarding *β*-glucosidase [59].

**Figure 8:**
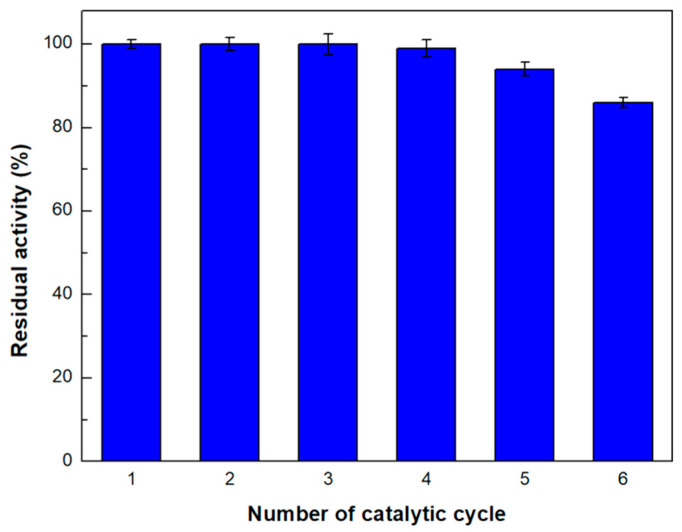
Operational stability of immobilized *β*-glucosidase in the multi-channel parallel micro-reactor. The value of 100% indicates the enzyme activity in the first catalytic cycle.

Furthermore, the reusability of the multi-channel microreactor was investigated for six successive catalytic cycles. As seen in Figure 8, the microreactor retains over 80% of its initial enzymatic activity, indicating that this configuration could be effectively applied for continuous catalytic cycles.

## 4. Conclusions

In this work, a single-channel microreactor and a multi-channel parallel microreactor were designed and fabricated. *β*-Glucosidase from *Thermotoga maritima* was immobilized in both microreactors. The single-channel microreactor was utilized to determine the catalytic properties of the enzyme; the immobilized enzyme demonstrated high operational stability, being able to be reused for up to nine reaction cycles while preserving more than 80% of its initial activity. Furthermore, the enzyme exhibited great thermal stability while the optimal pH for enzymatic activity was determined to be pH 7.0. The effect of flow rate was investigated and it was revealed that for the tested flow rates, the Km(app) decreased as the flow rate increased, indicating the restriction of mass transfer phenomena and high stability of the enzyme–substrate complexation. Lastly, the multi-channel parallel reactor was used to increase the output of the system, achieving 0.074 μmol/min p-NP production (which is 4.6-fold higher than that observed in the single-channel microreactor). This system could retain more than 80% of its activity for six consecutive reaction cycles. In summary, in this work, we demonstrated that *β*-glucosidase can be immobilized successfully in 3D-printed microreactors modified with chitosan to be used for industrial applications. Thus, 3D printing technology can be combined with microfluidics for applications in the industrial sector that require large volumes of products. However, further characterization of the multi-channel microreactor is necessary to have a complete understanding of the capabilities of this system. Future research could focus on the effect of different flow rates on the system as well as on the optimization of the devices for maximal product production and correlation of the results of the single-channel microreactor with those obtained from its multi-channel counterpart.

## Figures and Tables

**Table 1:** Apparent Km values for the immobilized *β*-glucosidase in the single-channel microreactor, for different flow rates.

Flow Rate (μL/min)	Apparent Km (mM)
20	0.854 ± 0.151 ^a^
30	0.585 ± 0.054 ^b^
40	0.551 ± 0.041 ^b^
50	0.486 ± 0.039 ^b^

Means with different letters are statistically significant (*p* < 0.05).

## Data Availability

Data are contained within the article.
